# Primiparous Adaptation with Postpartum Health Issues in Jeddah City, Kingdom of Saudi Arabia: A Quantitative Study

**DOI:** 10.3390/nursrep11040074

**Published:** 2021-10-12

**Authors:** Ahlam Al-Zahrani, Wedad Almutairi, Howaida Elsaba, Sanaa Alzahrani, Shouq Alzahrani, Linah Althobaiti, Ohoud Turkestani

**Affiliations:** 1 Maternity and Child Department, Faculty of Nursing, King Abdulaziz University, Jeddah 21589, Saudi Arabia; walmutairi@kau.edu.sa (W.A.); sanaa8mz@gmail.com (S.A.); shouqmalzahrani@gmail.com (S.A.); lalthobaiti0001@stu.kau.edu.sa (L.A.); ohoudtur@icloud.com (O.T.); 2 Maternity, Obstetric and Gynecological Nursing Department, Faculty of Nursing, Port Said University, Port Said 32223, Egypt; dr_howaidaamin@yahoo.com

**Keywords:** postpartum, health issues, women, depression, weight gain, adaptation, puerperium, Saudi Arabia

## Abstract

*Background*: The postpartum or puerperium period is the first 6 weeks after giving birth to an infant. The postpartum period can have negative implications, especially in first-time mothers. With their transition into motherhood, new mothers adopt new lifestyles, which can affect their physical wellbeing. Childbirth has physical, psychological, and emotional effects on women as they try to adapt to their new roles in order to get through this period with no or minimal health issues. *Study Aim*: The current study aims to explore primiparous adaptations with postpartum health issues in Jeddah City at Kingdom of Saudi Arabia. *Methods*: The research design is quantitative cross-sectional. A structured questionnaire was developed to collect data in relation to depression and weight gain, which consider the most common postpartum health issues. The inclusion criteria of the participants are: primipara, 2 to 6 months postpartum, and living in Jeddah. *Results*: 140 participants were included in the study. Mothers gained approximately 9.2 kg within the fifth to ninth month after giving birth. *Discussion*: Postpartum weight retention is a primary challenge in the majority of primiparous mothers and results in reduced quality of life. Nurses were always available to answer questions related to the postpartum health issues and explained the expectations to the family members. *Conclusions*: Childbirth and the postpartum period for first-time mothers are crucial in their lives as they try to adapt to a new way of life. The postpartum period can have negative implications, especially in first-time mothers. With their transition into motherhood, new mothers adopt new lifestyles, which can affect their physical wellbeing. More research is needed to explore the impact of postpartum health issues in Saudi Arabia.

## 1. Introduction

The postpartum period can have negative implications, especially in first-time mothers. With their transition into motherhood, new mothers adopt new lifestyles, which can affect their physical wellbeing. Childbirth has physical, psychological, and emotional effects on women as they try to adapt to their new roles in order to get through this period with no or minimal health issues [1]. Roy’s model defines adaptation as behavioral and cognitive efforts that a person makes to meet environment demands [2]. Factors in adaptation associated with the postpartum period include social support, educational level, the nursing care provided to new mothers, cultural and traditional practices, and physical exercise [3].

Social support has a great impact on both the physiological and psychological consequences of this critical period. At this stage, most mothers need support from their partners or their family members to avoid “baby blues” or other postpartum complications that can harm their health [4,5]. A Saudi study that determined the prevalence of postpartum depression in Riyadh found a significant association between the occurrence of postpartum depression (PPD) and an unsupportive spouse [6]. New mothers can suffer from anxiety and develop a feeling of maternal inadequacy if they lack adequate social support [7].

A high level of knowledge and education makes adaptation easier. A study showed that women who receive postpartum education show better postpartum adaptation [8]. In addition, nurses have a great impact on the care provided to a mother and her family. An important aspect of nursing is providing culturally based health education programs during pregnancy and the postnatal period that help mothers to overcome their fears and concerns [9,10].

Postpartum weight retention is the difference between weight after delivery and weight prior to pregnancy. Excessive weight retention as a result of hormonal and metabolic changes is a fundamental problem among women after giving birth to children. New mothers tend to ignore their health and wellbeing; they seldom have free time to engage in physical exercise, which is essential for regaining a healthy weight and promoting mental wellbeing; such exercise helps new mothers to more easily adapt to their new lives [11,12,13]. A nationwide cross-sectional survey, conducted by Althumiri et al. [14] has reported that the national weighted prevalence of obesity (BMI ≥ 30) was 24.7% in Saudi Arabia. They conclude that there is need for action to focus more on obesity in the Kingdom of Saudi Arabia.

First-time mothers commonly experience stress and depressive symptoms in the postpartum period. Many women find it difficult to manage the physical, social, and psychological challenges that accompany early motherhood [15]. Postpartum depression affects approximately 10–15% of Saudi mothers [16]. Some new mothers may have difficulty in exercising personal control and in accepting maternal responsibility, which can lead to postnatal depression [17]. In addition, during this critical period for caregiving behavior, parents experience changes in their sleep that may affect their ability to provide sensitive care [18]. Therefore, it becomes essential to explore the postpartum health issues that mothers experience in relation to depression and weight retention, especially in Jeddah, Saudi Arabia.

There is a need for investigating the postpartum health issues in communities with distinctive cultural values, such as the Kingdom of Saudi Arabia. Saudi community is a conservative and with strong influence of family and religion. Therefore, due to the limited existing literature around postpartum health issues within the Saudi community further research is warranted. The current study aimed to explore primiparous adaptations regarding postpartum health issues in Jeddah city, and the health issues studied were weight retention and depressive symptoms.

## 2. Materials and Methods

### 2.1. Study Design

A quantitative descriptive design was utilized to explore primiparous adaptations regarding postpartum health issues in Jeddah city. Descriptive designs allow the researcher to measure variables and provide opportunity to describe relationships between them.

### 2.2. Study Participants

A purposive sampling approach was used to gain access to study participants. The estimated required sample size was 140 participants, at a confidence level of 98% and a precision of 0.05 according to the Steven equation [19]. Therefore, 145 women who met the inclusion criteria (primipara, 2–6 months postpartum, live in Jeddah city and agree to participate in the study) were recruited to account for dropouts; eventually five were excluded for missing the exercise questions related to the weight retention measurements. Finally, 140 participants were included for further analysis.

### 2.3. Data Collection

The data were collected through an electronic survey due to the outbreak of COVID-19. The survey was distributed to a wide range of social media such as Facebook and WhatsApp to reach the study sample. Primipara mothers who live in Jeddah city and 2–6 months postpartum were invited to participate in the study. Jeddah City is the second largest city in the Kingdom of Saudi Arabia. It is located in the middle of the eastern coast of the red sea. In addition, it is one of the largest economic and commercial center in Makkah province, with population of about 4,697,000 people.

### 2.4. Study Tool

The study used a questionnaire that consisted of three parts. The first part aimed to collect information on sociodemographic characteristics, including general information (e.g., age, marital status, place of home, income, employment status, type of job, the level of education of the participant and her husband, father, and mother) and information on postpartum experiences (birth type, heath problem during birth, medical and contraception-use histories, gender of the newborn, duration of the postpartum period, postpartum difficulties, type of newborn feeding, having house made, and postpartum follow up). The second part aimed to collect information related to the weight retention of the participants (height, weight before pregnancy and after birth, physical exercises, and use of herbs to reduce weight).

The third part was the Edinburgh Postnatal Depression Scale (EPDS [20], a 10-item instrument developed for use by both pregnant and postpartum women to measure the risk of perinatal depression. The items include “I have been able to laugh and see the funny side of things” and “I have felt sad and miserable.” The instrument requires participants to indicate the response closest to how they have felt in the past seven days; the response labels vary across the items, but all range from 0 to 3. The scores are summed (with five reverse-scored items corrected) to obtain a total score. A score greater than 13 indicates a high likelihood of depression. The EPDS has been used in previous studies, and scores derived from the EPDS have demonstrated evidence of internal consistency [21,22]. The internal consistency estimates (α) for the EPDS scores in this study were in the range of 0.82–0.89 (M = 0.85, SD = 0.03).

### 2.5. Bias

It is likely that no research can be totally free from possible bias, but researchers should work hard to identify and control all sources of bias to deliver the highest-quality of research as possible. For the current study the researchers controlled the non-response bias by using a short and simple online survey which helped in distributing the survey to increase the participants’ response. A quantitative design was used to collect responses to control any events that might affect women’s responses. Data collection bias was controlled by entering the data carefully into the Statistical Package for Social Sciences program (SPSS) version 20.0 (Armonk, NY, USA, IBM Corp), then checking the entries twice by one of the researchers to avoid any potential bias.

### 2.6. Variables

The independent variable is a singular characteristic that cannot be change or manipulated by the researcher such as age, sex and ethnicity. While the dependent variables rely on and can be changed by factors such as weight and depression. It is known that the independent variables can have influence on the dependent variables. Therefore, in the current study the researchers take into account the independent variable which is the socio-demographic characteristics for the study participants and the dependent variable which are the postpartum health issues that include weight retention and depression to explore any relation between variables.

### 2.7. Data Analysis

The data were analyzed using the IBM SPSS software package version 20.0 (Armonk, NY, USA, IBM Corp). Quantitative data are represented as means and standard deviations and are presented as numbers and percentages. Descriptive statistics and tests of significance were used for testing the association of variables difference in the study. All data was represented in tables.

### 2.8. Ethical Consideration

The research proposal was reviewed and approved by the ethical committee of the Faculty of Nursing at King Abdulazaiz University (Ref. No. 2B.1). In order to address ethical considerations aspect of the current study, the researchers obtained consent from the participants prior to the study which was requested at the beginning of the online survey. Anonymity of the women participating in the research was also ensured as no name was required in the survey. The reporting of the current research is consistent and complaint with STROBE reporting guidelines for observational research.

## 3. Results

A total of 140 primiparous mothers responded completely. Table 1 shows the sociodemographic characteristics of the participants: 55.7% of the participants were aged 31 years and above, 42% were 20–30 years old, and 1.4% were less than 20 years old. A proportion of 97.9% were married, and 2.1% were divorced. The data show that 47.1% of the participants lived away from their and their husband’s families, 37.1% lived near their husband’s family, 14.3% lived near their family, and 1.4% lived near both their and their husband’s families. A total of 51.4% of the participants had monthly incomes higher than 10,000 Saudi riyals; 58.6% of the women were employed, and 40.2% were teachers.

Table 2 shows the educational levels of the participants and their husbands, mothers, and fathers. The data show that 92.1% of the participants held university/postgraduate degrees, 7.1% had elementary/middle/high school degrees only, and only one participant (0.7%) said “other.” The majority (75%) of the participants’ husbands held university/postgraduate degrees, while 25% of the husbands had elementary/middle/high school degrees only. A total of 40.7% of the participants’ mothers had elementary/middle/high school degrees only, 40.7% were illiterate/could not read or write, and 18.6% had university/postgraduate degrees. Of the participants’ fathers, 53.6% had elementary/middle/high school degrees only, 29.3% had university/postgraduate degrees, and 17.1% were illiterate/could not read or write.

Table 3 presents the postpartum experiences of the new mothers: 59.3% of the mothers were 5–6 months postpartum, 66.4% had their children through spontaneous vaginal delivery, and the other 33.6% had cesarean sections. Additionally, 56.4% of the mothers had health issues during childbirth: 42.9% suffered from delayed labor, 38.6% suffered from the umbilical cord wrapping around the baby, and 28.6% suffered heavy bleeding. A total of 78.6% followed up with the physician after giving birth. The majority (79.3%) had no prior medical history. Housemaids were employed by 50.7% during the postpartum period, and the other 49.3% had no housemaids. Of the mothers, 41.4% spent 40 days of their postpartum period away from their homes. The majority spent their postpartum periods at their family’s house (56.4%), while 42.8% spent them at their husband’s family’s house. An inability to sleep was suffered by 52.1% of the mothers, 13.6% had adaption problems, 13.6% suffered from depression, and 8.6% suffered from an increase in weight. Lastly, 6.4% found it difficult to deal with the baby crying and breastfeeding, and 63.6% fed their babies by both breastfeeding and bottle.

Table 4 shows the data related to postpartum weight retention. The average height of the participants was 157.6 cm. The average pre-pregnancy weight was 60.6 kg, the average pregnancy weight was 60.6 kg, and the average current weight was 69.4 kg. Herbs and medications were used by 30.7% of the participants to help them lose weight faster, while the other 69.3% did not use any herbs or medications. A total of 50.7% exercised, and of those, the majority walked (34.4%), while 20% did various workouts in the gym and 3.6% worked out in their homes. Of the participants, 79.3% were concerned about their weight, while the other 20.7% were not concerned about their weight, and 70.7% did not follow a specific diet. The majority ate three meals a day (42.1%), while 19.3% and 38.6% ate more and fewer than three meals/day, respectively. A total of 72.1% did not feel pressure from society about their weight, while 27.9% did feel such pressure from society; 59.3% were not satisfied with their weight, while 40.7% were satisfied. A total of 46.4% were expecting to return to pre-pregnancy weight within three months, while 53.6% were not. 

Table 5 shows the scores for the Edinburgh Postnatal Depression Scale (EPDS) items among the study participants; the mean number of participants able to laugh and see the funny side of things was 2.56 ± 0.626.

Table 6 shows that nurses’ roles during the postpartum period can help the mothers adapt to postpartum health issues effectively. The mean satisfaction with the information provided at the hospital regarding the expected postpartum problems was 2.11 ± 0.814.

It is clear in Table 7 that there was a positive direct relationship between social support and depression after childbirth (Pearson’s correlation coefficient = 0.717; Table 7), which means that, if there was social support, then the health condition improved more quickly. There was also a positive correlation between depression and weight gain (Pearson’s correlation coefficient = 0.850).

## 4. Discussion

The study participants’ demographics played an important role in explaining the differences in the results. Most of the primiparous mothers were aged 20 years and above and were financially stable, earning over SR 10,000 per month. Therefore, most of the mothers were of legal age and did not have financial difficulties, which is a notable challenge for primiparous mothers worldwide [23]. The majority of the mothers were also married and lived with their husbands or their families; hence, the availability of physical support was not a problem. Most of the primiparous mothers had careers that required high involvement, including being teachers, nurses, and physicians. High-involvement careers could be a source of anxiety due to job pressure [24]. The main challenges faced by primiparous mothers, as well as the contributing factors, are discussed below.

One of challenges faced by primiparous mothers is postpartum weight retention, a primary challenge for the majority of primiparous mothers, which results in a reduced quality of life. According to the study, approximately 59.3% of the mothers were dissatisfied with their weight after giving birth. Only a small group of the mothers took the initiative in the management of their weight. Regardless of their concerns about weight gain, most did not take any supplements or participate in weight management activities such as physical activity or managing their diet, which may put them at risk for obesity-related diseases such as diabetes. Moreover, the average weight retention was 6 kg and majority of the sample have bachelor degree which contradicts the results of previous studies which founds higher education associated with lower average weight retention [25,26].

Weight gain has positive correlation with the depression symptoms which aligned with previous studies findings that found strong association between postpartum depression and postpartum weight retention which may explained by the common strategy women used to adopt with the psychological, emotional, or physical discomort during the postpartum period [27,28].

Nurses play a significant role not only in assisting mothers during delivery, but also in the provision of medical and psychological support in the postpartum period. Nurses’ explanations of the postpartum period were sufficient and played a significant role in explaining their expectations. Moreover, nurses were always available to answer questions related to the period and explained the mother’s expectations to her family members. A sufficient explanation of postpartum challenges and how to manage them plays a significant role in upholding the quality of life associated with the postpartum period in primiparous mothers [29]. Importantly, a mother’s family members should make an effort to understand the challenges and expectations during the postpartum period, which was adequately facilitated.

Education levels affect mothers’ levels of understanding and interpretation of knowledge. Nurses and other medical practitioners could play a remarkable role in enlightening patients about their expectations, but without an appropriate level of understanding, the information may not serve any purpose [30]. According to this study, 99.3% of the participants had qualifications above elementary education, as did all of their husbands. Their parents were also well educated, with a significant number reporting that they could read and write.

The postpartum experience of new mothers significantly influences their quality of life, as well as their willingness to address their health problems and have another child. The majority of the mothers experienced normal vaginal births, while a small number had cesarean sections. Most of the mothers also reported having faced different challenges during delivery, including bleeding, inappropriate umbilical cord positioning, and delayed labor. Many reported challenges during childbirth, including tissue straining and surgical procedures, that led to a reduced quality of life after the process [31]. Therefore, giving birth and the quality of life led after the postpartum period play a significant role in the lifestyles of the primiparous mothers and their health outcomes.

According to the study’s findings, the majority of the mothers faced health challenges during the postpartum period due to weight gain and other issues. The majority had significant weight gain, which poses a risk to their health. Childbirth and the postpartum period are crucial in the lives of first-time mothers as they try to adapt to a new way of life. Primiparous mothers must manage this stage with limited or no health issues. Successfully getting through the postpartum period depends on the available resources and stressors. To meet the new demands, primiparous mothers have to adapt through cognitive and behavioral efforts. Some of the adaptation factors for primiparous mothers include exercise, social support, cultural and traditional practices, educational level, and nursing care. In contrary to this study, high level of anxiety and depression was found in another Saudi study using the same instrument [6]. In Saudi Arabia, women experience postpartum depression which affects their health. The psychological and physiological challenges for first-time mothers can be minimized through social support, so offering them such support is important.

According to this study, postpartum education also plays a significant role in ensuring that first-time mothers adapt to the new challenges following childbirth. The findings revealed that mothers who receive postnatal training adapt more quickly than those who do not. Moreover, nursing care is also a critical factor for primiparous adaptation. Mothers are helped to overcome their fears and concerns through health education programs provided by nurses. Nurses also help mothers to assess family needs during the postpartum stage. Cultural and traditional factors also influence the postpartum period. Lastly, this study showed the significance of exercise during the postpartum period; this practice engages the mothers, thus reducing the likelihood of depression and also helps with weight retention, improves muscle strength, and boosts self-energy. As they adapt to providing care to their newborn, their body weight is affected. Therefore, incorporating exercise is important in the management of the postpartum period.

## 5. Limitations

The outbreak of COVID-19 was the main limiting factor in this study. Due to the isolation guidelines and the pandemic, the data were collected through an electronic survey, which limited the number of participants. Additionally, the findings cannot be generalized because of the small sample size, which might affect the representation of the study population.

## 6. Conclusions

Successfully getting through the primiparous postpartum period depends on the social support, exercise, educational level, and nursing care provided to mothers. The other challenges these mothers face include weight retention and postpartum depression. First-time mothers need access to education and training from nurses to assist them in adapting to the new life of motherhood. During this training, nurses should address all of the fears and concerns raised by mothers. They should also clarify all of the myths of childbirth and provide factual information. Nurses are responsible for educating mothers on various aspects of attending to their newborn, nutrition, and general health practices. Nurses also address all of the concerns mothers could raise and thus improve their confidence in caring for their children.

During the postpartum period, mothers should have exercise schedules that will help in weight management. Exercise also keeps mothers busy, thus reducing the chances of depression. Since educational levels and nursing experience are not a significant cause of primiparous mothers’ poor postpartum life experiences, relevant authorities should work on managing the experience of giving birth as the primary cause of reduced quality of life in primiparous mothers. The implementation of initiatives that will reduce poor health outcomes during delivery, such as the management of pain and complications, is recommended. Finally, it is recommended that family, friends, and medical practitioners offer primiparous social support. This support is critical in helping mothers to adapt to motherhood and to avoid suffering from depression.

## Figures and Tables

**Table 1:** Sociodemographic characteristics of the study participants.

Sociodemographic Characteristics	*n*	%
Age:		
• Less than 20 years	2	1.4
• 20–30 years	60	42
• 31 years or more	78	55.7
Marital status:		
• Married	137	97.9
• Divorced	3	2.1
Place of home:		
• Near to their family	20	14.3
• Near to their husband’s family	52	37.1
• Away from both families	65	47.1
• Near to both families	3	1.4
Income:		
• Less than 5000 Riyals	9	6.4
• 5000–10,000 riyals	59	42.1
• More than 10,000 Riyals	72	51.4
Employment status:		
• Employed	82	58.6
• Housewife	58	41.4
Type of job:		
• Teacher	33	40.2
• Nurse	22	26.8
• Physician	22	26.8
• Administrative	5	6.2

**Table 2:** Educational levels of the participants and their husbands, mothers, and fathers.

Educational Level	*n*	%
The participants:		
• University/postgraduate	129	92.1
• Elementary/middle/high school	10	7.1
• Other	1	0.7
The husbands:		
• University/postgraduate	105	75
• Elementary/middle/high school	35	25
The mothers:		
• University/postgraduate	26	18.6
• Elementary/middle/high school	57	40.7
• Illiterate/could not read or write	57	40.7
The fathers:		
• University/postgraduate	41	29.3
• Elementary/middle/high school	75	53.6
• Illiterate/could not read or write	24	17.1

**Table 3:** Postpartum experiences for the new mothers.

Experience Items	*n*	%
Birth type:		
• Spontaneous vaginal delivery	93	66.4
• Cesarean section	47	33.6
Health problems during birth:		
• Yes	79	56.4
• No	61	43.6
Health problems during birth:		
• Bleeding	26	28.6
• Umbilical cord wrapped around the baby	26	28.6
• Delayed labor	39	47.1
Past medical history:		
• Lung disease	9	6.4
• Heart disease	4	2.9
• Diabetes	9	6.4
• Blood pressure problems	7	5
• No past medical history	111	79.3
Where did you spend the postpartum period?		
• Your house	58	41.5
• Your family’s house	79	56.4
• Your husband’s family’s house	3	2.1
Postpartum period duration:		
• Less than one month	30	21.4
• One month	31	22.1
• 40 days	58	41.4
• More than 40 days	21	15
Did you have a housemaid?		
• Yes	71	50.7
• No	69	49.3
How di you feed the baby?		
• Bottle feeding only	30	21.4
• Breastfeeding only	21	15
• Breastfeeding and bottle feeding	89	63.6
Postpartum period difficulties:		
• Anxiety	8	5.7
• Inability to sleep	73	52.1
• Depression	19	13.6
• Adaptation problems	19	13.6
• Weight increasing	12	8.6
• Baby crying	9	6.4
Did you follow up with the physician?		
• Yes	110	78.6
• No	30	21.4

**Table 4:** Data related to postpartum weight retention among the study participants.

Items	Frequency (*n* = 140)	Percent or Mean ± SD
Height	140	157.6 ± 15.3
Pre-pregnancy weight	140	60.6 ± 14.2
Current weight	140	69.4 ± 18.5
Use of herbs or medication to lose weight:		
-Yes	43	30.7%
-No	97	69.3%
Exercise:		
-Yes	81	57.9%
-No	59	42.1%
Type of exercise (*n* = 81):		
-Walking	48	34.3%
-Gym	28	20%
-Workout at home	5	3.6%
Concerned about weight:		
-Yes	111	79.3%
-No	29	20.7%
Did you follow a specific diet?		
-Yes	99	70.7%
-No	41	29.3%
Number of meals:		
-Fewer than 3 meals	54	38.6%
-3 meals	59	42.1%
-More than 3 meals	27	19.3%
Pressure from society:		
-Yes	39	27.9%
-No	101	72.1%
Satisfied with your weight?		
-Yes	57	40.7%
-No	83	59.3%
Expected weight gain after birth:		
-Yes	123	87.9%
-No	17	12.1%
Expected to return to pre-pregnancy weight in 3 months:		
-Yes	65	46.4%
-No	75	53.6%

**Table 5:** Results for Edinburgh Postnatal Depression Scale (EPDS) items among the study participants.

EPDS Items		Mean ± SD
1	I feared or panicked for no good reason after birth	1.96 ± 0.812
2	I was worried for no good reason after birth	1.97 ± 0.822
3	I was looking forward to coming while enjoying things after birth	2.46 ± 0.713
4	I managed to laugh and see the funny side of things after birth	2.56 ± 0.626
5	I was unnecessarily blaming myself when things went wrong after birth	1.79 ± 0.803
6	I accumulated work, so I couldn’t do it after birth	1.90 ± 0.851
7	I was so unhappy that I had trouble sleeping after birth	1.70 ± 0.756
8	Did you feel sad or unhappy after giving birth?	1.48 ± 0.683
9	I had in mind the idea of harming myself after giving birth	1.11 ± 0.410
10	I was so unhappy that I was crying after birth	1.57 ± 0.788

**Table 6:** Nurses’ roles during the postpartum period as mentioned by the study participants.

Nurses’ Roles	Mean ± SD
The nurse’s explanation of postpartum problems and what is expected to happen was clear	1.91 ± 0.804
The nurse was available to answer inquiries regarding expected postpartum problems	1.95 ± 0.892
The nurse informed the family about my condition and my expected needs for postpartum problems	1.74 ± 0.886
The nurse allowed me to inquire about expected postpartum problems	2.08 ± 0.857
I was satisfied with the information provided at the hospital regarding the expected postpartum problems	2.11 ± 0.814

**Table 7:** Relations between Weight Gain, Depression and Social support.

		Weight Gain	EPDS	Social Support
Weight Gain	Pearson’s correlation	1	0.850	0.633
	Sig. (2-tailed)		0.558	0.697
	*n*	140	140	140
EPDS	Pearson’s correlation	0.850	1	0.617
	Sig. (2-tailed)	0.558		0.842
	*n*	140	140	140
Social Support	Pearson’s correlation	0.633	0.717	1
	Sig. (2-tailed)	0.697	0.842	
	*n*	140	140	140

## Data Availability

The data presented in this study are available on request from the first author.
